# Reservoir Competence of Vertebrate Hosts for *Anaplasma phagocytophilum*

**DOI:** 10.3201/eid1812.120919

**Published:** 2012-12

**Authors:** Felicia Keesing, Michelle H. Hersh, Michael Tibbetts, Diana J. McHenry, Shannon Duerr, Jesse Brunner, Mary Killilea, Kathleen LoGiudice, Kenneth A. Schmidt, Richard S. Ostfeld

**Affiliations:** Bard College, Annandale-on-Hudson, New York, USA (F. Keesing, M.H. Hersh, M. Tibbetts, D.J. McHenry);; Cary Institute of Ecosystem Studies, Millbrook, NY, USA (F. Keesing, M.H. Hersh, S. Duerr, R.S. Ostfeld);; Washington State University, Pullman, Washington, USA (J. Brunner);; New York University, New York, New York, USA (M. Killilea);; Union College, Schenectedy, NY, USA (K. LoGiudice);; Texas Tech University, Lubbock, TX, USA (K.A. Schmidt)

**Keywords:** Anaplasma phagocytophilum, bacteria, reservoir competence, vertebrate hosts, bacteria, infectivity, prevalence, tick-borne diseases, ticks, zoonoses

## Abstract

Fourteen vertebrate species (10 mammals and 4 birds) were assessed for their ability to transmit *Anaplasma phagocytophilum*, the bacterium that causes human granulocytic anaplasmosis, to uninfected feeding ixodid ticks. Small mammals were most likely to infect ticks but all species assessed were capable of transmitting the bacterium, in contrast to previous findings.

Human granulocytic anaplasmosis (HGA), formerly known as human granulocytic ehrlichiosis, is an emerging infectious disease in the United States, Europe, and Asia (*1*,*2*). In the United States, most reported cases are concentrated in north-central and northeastern states. Patients with HGA typically have nonspecific febrile symptoms, including fever, chills, headache, and myalgia (*1*). Most patients with HGA respond well to antimicrobial drug treatment, but complications are not uncommon and some cases are fatal (*2*). Because of difficulties in diagnosis and lack of awareness of HGA by physicians and the public, many cases are misdiagnosed, and national statistics likely dramatically underreport this disease (*1*).

HGA is caused by a rickettsial bacterium, *Anaplasma phagocytophilum* (*1*), groups of which form dense aggregations in granulocytes (*3*). The bacterium is passed from host to host through the bite of an infected ixodid tick: *Ixodes scapularis* in the eastern and central United States and *Ix. pacificus* in the western United States (*4*–*6*). Serosurveys and molecular diagnostics within disease-endemic zones show that many ground-dwelling vertebrate species are exposed to or infected with *A. phagocytophilum* (*2*). These data indicate that tick-to-host transmission rates are high and that infection is widespread in host communities.

However, few studies have examined rates of transmission from infected hosts to uninfected ticks, a trait known as the reservoir competence of these hosts. Quantification of host species–specific reservoir competence can identify animals most responsible for producing infected ticks and therefore increasing risk for human exposure. Overall, robust quantitative information on reservoir competence is scarce and key hosts remain unstudied. We determined the reservoir competence for *A. phagocytophilum* of 14 species (10 mammals and 4 birds) in a disease-endemic region of the eastern United States.

## The Study

All procedures were conducted after approval from the Cary Institute of Ecosystem Studies Institutional Animal Care and Use Committee. We conducted our research in Dutchess County, New York, a region where human cases of anaplasmosis are rapidly increasing. We trapped hosts on the property of the Cary Institute of Ecosystem Studies (Millbrook, NY, USA) during the peak abundance of larval blacklegged ticks (*Ix. scapularis*) during July–September in 2008, 2009, and 2010. Detailed methods have been reported (*7*). 

We held members of 10 mammal and 4 bird species (Table 1) for 3 days in cages with wire mesh floors suspended over pans lined with wet paper towels. Ticks feeding on hosts were allowed to feed to repletion and drop from hosts into the pans, from which they were collected. In some cases, if hosts did not drop >10 ticks within 3 days, we infested them with unfed larval ticks following methods described (*8*). Because no evidence has been found for transovarial transmission of *A. phagocytophilum* (*9*) or of infection in larval ticks, these infestations likely did not affect host exposure to the pathogen. Hosts that had been infested were held for an additional 4 days, and engorged ticks were collected each day. All engorged larval ticks were held in moistened glass vials at constant temperature and humidity until they molted into the nymphal stage. Newly molted nymphs were flash-frozen in liquid nitrogen and stored at −80°C.

**Table 1:** Host species tested for *Anaplasma phagocytophiluum* reservoir competence, southeastern New York, USA, 2008–2010*

Host species	Common name	No. hosts tested	No. ticks tested	Mean no. ticks sampled per host (range)
Mammals				
* Blarina brevicauda*	Northern short-tailed shrew	28	529	18.9 (11–25)
* Didelphis virginiana*	Virginia opossum	25	501	20.0 (11–25)
* Glaucomys volans*	Southern flying squirrel	4	59	14.8 (6–25)
* Mephitis mephitis*	Striped skunk	1	21	21.0 (21–21)
* Peromyscus leucopus*	White-footed mouse	30	571	19.0 (10–25)
* Procyon lotor*	Raccoon	25	484	19.4 (10–25)
* Sciurus carolinensis*	Eastern gray squirrel	20	358	17.9 (10–25)
* Sorex cinereus*	Masked shrew	6	41	6.8 (4–10)
* Tamias striatus*	Eastern chipmunk	19	300	15.8 (9–25)
* Tamiasciurus hudsonicus*	Eastern red squirrel	15	297	19.8 (11–25)
Birds				
* Catharus fuscescens*	Veery	21	427	20.3 (10–25)
* Dumetella carolinensis*	Gray catbird	14	235	16.8 (9–24)
* Hylocichla mustelina*	Wood thrush	28	496	17.7 (10–24)
* Turdus migratorius*	American robin	18	321	17.8 (8–24)

*Number of ticks tested per host can include samples from either natural body loads or experimental infestations, as described in the text, and is not representative of mean total body loads.

DNA extraction was conducted as described (*7*). To amplify extracted DNA, we used protocols reported by Courtney et al. (*10*). Briefly, we used primers ApMSP2f and ApMSP2r and probe ApMSP2p, which are specific for the *msp2* gene of *A. phagocytophilum* and generate a 77-bp fragment. Real-time PCR was performed by using a CFX96 Real-Time PCR System (Bio-Rad, Hercules, CA, USA) . We used extracted DNA from unfed larval ticks and ultrapure water as negative controls to account for potential contamination during the extraction and PCR processes, respectively. The cloned 77-bp fragment was used as a positive control. Barrier pipette tips were used throughout the process to prevent contamination. We conducted 3 replicate PCRs per tick.

Ticks were considered positive for *A. phagocytophilum* if any 1 of 3 replicate samples showed amplified DNA for *A. phagocytophilum* relative to negative controls. Ticks with marginal results (i.e., moderate fluorescence) were tested a second time with the same primers and SYBR green dye. For these confirmatory tests, we included a melt curve analysis in which we determined the temperature at which half of the PCR products had denatured. PCR products were heated from 70°C through 85°C, raising the temperature by 0.5°C every 10 s. Positive controls consistently had melting point maxima of 80.5*°*C. Using a TOPO-TA Cloning Kit (Invitrogen, Carlsbad, CA, USA), we cloned and sequenced 140 fragments that had a melting point of 80.5*°*C. Identity of sequences was confirmed by conducting BLAST searches (National Center for Biotechnology Information, Bethesda, MD, USA) of GenBank using the blastn algorithm (*11*). One hundred thirty-one of 140 fragments were identified as *A. phagocytophilum*; the remaining 9 fragments either had poor-quality sequences or did not have the cloning vector inserted. If any replicate was positive in the confirmatory test, ticks were considered positive for *A. phagocytophilum*. If all 3 replicates in the confirmatory test showed marginal or negative results, the ticks were considered negative. Reservoir competence for each host species was calculated as the average percentage of ticks infected per individual host.

**Figure 1:**
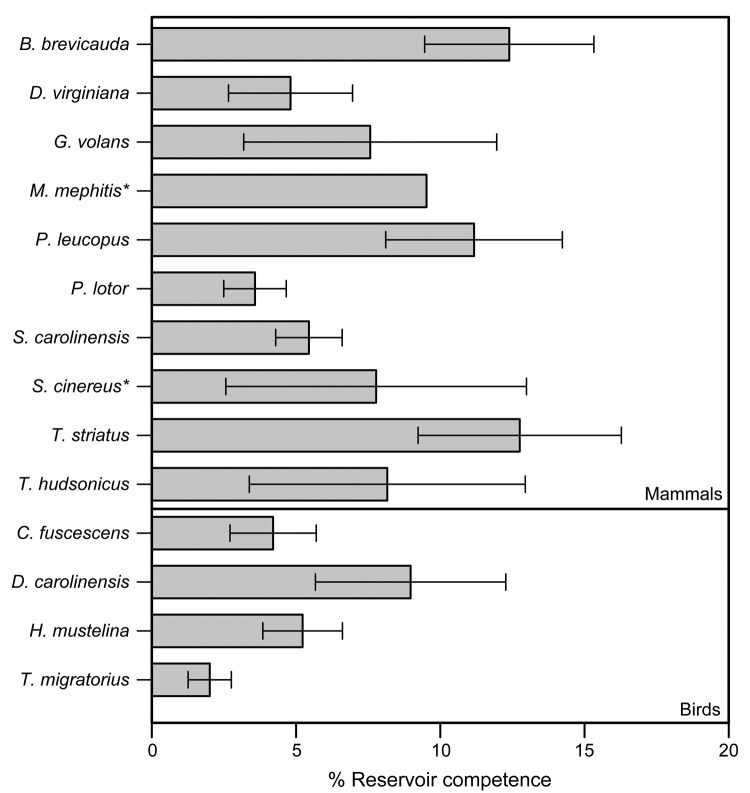
Mean reservoir competence of 14 host species (10 mammals and 4 birds) for *Anaplasma phagocytophilum*, southeastern New York, USA, 2008–2010. Error bars indicate SE. Reservoir competence is defined as the mean percentage of ticks infected by any individual host of a given species. Host species with <10 individual hosts sampled are indicated by an asterisk. See Table 1 for sample sizes. Single-letter abbreviations for genera along the left indicate *Blarina*, *Didelphis*, *Glaucomys*, *Mephitis*, *Peromyscus*, *Procyon*, *Sciurus*, *Sorex*, *Tamias*, *Tamiasciurus*, *Catharus*, *Dumetella*, *Hylocichla*, and *Turdus*, respectively.

**Figure 2:**
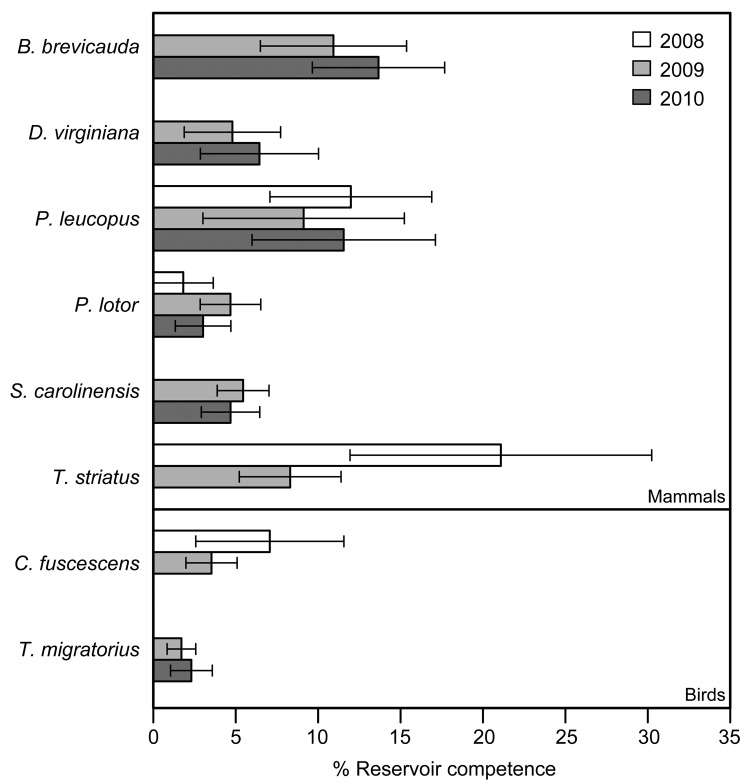
Mean reservoir competence of 14 host species (10 mammals and 4 birds) for *Anaplasma phagocytophilum*, southeastern New York, USA, 2008–2010. Error bars indicate SE. Reservoir competence is defined as the mean percentage of ticks infected by any individual host of a given species. For inclusion, sample sizes for a species had to be >4 in >2 years. No species showed significant variation in reservoir competence across years (p>0.10, by 2-way analysis of variance or Kruskal-Wallis test as appropriate, for all species tested). Single-letter abbreviations for genera along the left indicate *Blarina*, *Didelphis*, *Peromyscus*, *Procyon*, *Sciurus*, *Tamias*, *Catharus*, and *Turdus*, respectively.

Using data for 4,640 ticks collected from 254 animals over 3 years, we assessed levels of reservoir competence for 14 host species (10 mammals and 4 birds) (Table 1). Short-tailed shrews, white-footed mice, and eastern chipmunks had mean levels of reservoir competence >10% (Figure 1). All other hosts, including opossums, gray and red squirrels, and all 4 species of birds, had mean levels of reservoir competence ranging from 2% to 10%. Reservoir competence differed significantly among these 11 species (*F* = 2.294, df = 10,232, p = 0.014, by 2-way analysis of variance). Southern flying squirrels, striped skunks, and masked shrews all transmitted *A. phagocytophilum* to ticks, but our sample sizes were too small to draw strong conclusions about reservoir competence. For species that we collected in abundant numbers in multiple years (>4 animals in >2 years), reservoir competence of each species did not vary significantly from year to year (p>0.10 for all species tested, by analysis of variance or Kruskal-Wallis tests as appropriate) (Figure 2).

## Conclusions

Our data contradict several assumptions about the role of hosts in infecting ticks with *A. phagocytophilum*. First, the role of the white-footed mouse in infecting ticks has been controversial (*2*). Our data suggest that although the mouse is a major reservoir, short-tailed shrews and eastern chipmunks have comparable levels of reservoir competence. In addition, previous work has suggested that chipmunks, skunks, and opossums do not infect feeding ticks (*12*). At our sites, all of these species infected feeding ticks (Table 2). Thus, the potential for these hosts to contribute to human risk for HGA should not be ignored.

**Table 2:** Host species infected with *Anaplasma phagocytophilum* southeastern New York, USA, 2008–2010*

Host species	No. hosts infected/no. tested (%)	No. (%) ticks infected	Mean % infected ticks per infected host (range)
Mammals			
* Blarina brevicauda*	17/28 (61)	67 (13)	20 (4–56)
* Didelphis virginiana*	9/25 (36)	20 (4)	13 (4–50)
*Glaucomys volans*†	2/4 (50)	5 (8)	15 (14–16)
*Mephitis mephitis*†	1/1 (100)	2 (10)	10
* Peromyscus leucopus*	15/30 (50)	63 (11)	22 (4–50)
* Procyon lotor*	10/25 (40)	17 (4)	9 (4–20)
* Sciurus carolinensis*	14/20 (70)	19 (5)	8 (4–20)
*Sorex cinereus*†	2/6 (33)	4 (10)	23 (17–30)
* Tamias striatus*	10/19 (53)	40 (13)	24 (6–46)
* Tamiasciurus hudsonicus*	7/15 (47)	17 (6)	17 (4–73)
Birds			
* Catharus fuscescens*	9/21 (43)	19 (4)	10 (4–25)
* Dumetella carolinensis*	7/14 (50)	20 (9)	18 (4–33)
* Hylocichla mustelina*	14/28 (50)	27 (5)	10 (4–25)
* Turdus migratorius*	6/18 (33)	7 (2)	6 (4–11)

*Infected hosts are those that transmitted *A. phagocytophilum* to >1 *Ixodes scapularis* tick larva. †Host species with <10 individual hosts sampled.

Because hosts are capable of clearing *A. phagocytophilum* infections (*13*), surveys of host exposure might not represent species-specific probabilities of transmitting the pathogen to uninfected ticks. Instead, the role of particular species in contributing to the pool of infected ticks is best assessed by determining host reservoir competence using field-captured animals that usually carry ticks. On the basis of the community of hosts we sampled, small mammals are most responsible for infecting uninfected larval ticks in nature, and this result is consistent across years.
